# Aspects of plant intelligence (ii)

**DOI:** 10.1080/15592324.2026.2737116

**Published:** 2026-09-26

**Authors:** Anthony Trewavas

**Affiliations:** a Institute of Molecular Plant Science, University of Edinburgh, Edinburgh, Scotland

**Keywords:** Signal transduction, biological Intelligence, artificial intelligence, cognitive cell signaling, purposeful behavior, two gravitropic mechanisms, learning and memories

## Abstract

Plant intelligence was earlier described as adaptively variable behavior during the lifetime of the individual plant (Trewavas 2003). This general definition for animals and plants (biological intelligence) is now justified from generic terms and emphasis on adaptive behavior. Plant intelligence can also be defined from dictionary definitions of intelligence. Artificial intelligence has been indicated as adaptive behavior. Prominent psychological opinion now indicates that human intelligence is adaptive: IQ does not measure human intelligence. The uniformity of intelligence definitions negates views that plant intelligence is controversial. It is suggested that the early stages of transduction through calcium are interpreted through complex protein structures attached to mobile lipid rafts. Cell signaling acting as a cognitive process is investigated and evidence supports a relationship. Plants are biologically purposeful; they express goal-directed behavior throughout their life cycle and act as biologically purposeful agents in the wilderness. The demise of the Modern Synthesis of Evolution is indicated and could be replaced by one that correctly emphasizes biological purpose. Two sensory mechanisms are described for gravitropism as an illustration of the fail-safe mechanisms needed for all forms of behavior because true wilderness is often environmentally uncertain. Definitions of learning and memory are suggested and five kinds of plant biological memories are described.

## Introduction

In this article, plant behavior refers to the total of responses by plants to a change in a signal or stimulus. Many behaviors involve changes in development eventually altering visible growth (so-called tropic responses) or other morphological features. This article concentrates on those behaviors that are adaptive; that is they improve the probability of survival of plants living in wilderness.^1^ In Trewavas^2^ these behaviors were classed as intelligent using the definition of biological intelligence as “*Adaptively-variable behavior during the lifetime of the individual*”. Trewavas^2^ has been well-cited (currently 784, Google scholar) and it has generated considerable discussion in articles and books covering plant intelligence. Despite this degree of attention and debate much remains unsaid. Some regard intelligence as obvious while for other it remains apparently controversial.

This definition of plant intelligence, first suggested by Stenhouse^3^ in reference to animal evolutionary behavior, is later justified for plants that live in natural, wild circumstances. Intelligence describes the wide ability of plants to manage the complex, uncertain, and often threatening challenges of the natural world in which plants struggle to survive and reproduce. To a very much lesser extent, it can accommodate the agricultural and other human-manipulated environments whose environmental complexity is substantially reduced. Adaptive behaviors, or behavioral traits, are behaviors that improve the probability of survival.^1^ Adapt, or even acclimate, simply imply adjustment and do not replace the meaning and active implication of the word adaptive. Although adaptive capabilities can be investigated from laboratory research, such artifactual environments do not easily mimic the wilderness conditions in which biological intelligence contributes to differential survival. In my experience, the evolutionary context of “adaptive” is often not appreciated.

Some consider that the term intelligence for plants is subjective^4^ ^,^ ^5^ based on the supposition that IQ subjectively measures human intelligence. Years earlier, Robert Sternberg, the most prominent psychologist and prolific supporter of IQ as human intelligence, had concluded that IQ did not measure human intelligence at all.^6^ Sternberg^7^ now concludes that real human intelligence is adaptive behavior and identifies the skills required to manage survival in real-world contexts. IQ simply measures scholastic aptitude^8^ and is a psychological, not a biological, term. It has been described as a cultural artifact.^9^ IQ, via its associated tests, was originally designed to help identify children who needed special educational help. There is also an argument over whether IQs are related to individual human social advancement or other human benefit.^10^ IQ can only be used for one species: mankind. It cannot be used for the eight million or more species on earth.

When a zebra runs away from a lion, I regard that as intelligent behavior because it is adaptive. When I read that an ameba can immediately distinguish a *Euglena* cyst from a grain of sand of the same size and will devour the former while immediately rejecting the latter.^11^ (p. 18), I see that as intelligent too. And that is important because plants evolved from an amebic ancestor. Darwin's (1891, p. 95–96) observations on tendrils actively exploring post holes to act as a support also describe intelligence. When one hole is found, the tendril assesses the size (diameter?) of the hole by attempted insertion. If this is too small, it searches for an alternative until one of suitable size and depth is finally found.^12^ (p. 88). These examples of biological intelligence are each in their own, but different way adaptive; they improve survival probability.

### The problems of an animal framework for biology

The world we construct is the perceived world of an animal. The limitations of this perception for understanding plants are described elsewhere.^12^ (p. 14–16). I cannot avoid the fact that, being an animal, I have to use a language constructed by other members of my species. That may be a serious perceptual constraint in trying to understand plants. Many zoologists are intrigued by what they regard as the complexity of animal life. Using the criteria of complexity and comparing angiosperms with mammals, a definite conclusion could not be reached as to plants being simple and animals complicated.^12^ (p. 65–73). The angiosperm cell is likely as complicated as a mammalian cell or even more so. Understanding of either is still poor.

The difference between plant and animal behavior was clarified by Arber.^13^ Most plant behavior is really development. Cell proliferation and subsequent differentiation commence in an embryogenic meristem. Cell maturation occurs in distinct and elongating stem and root tissues. From a human perspective, this process is inevitably slow. But the equivalent in animal development is usually slower. The zygote, enclosed in a protected environment of the egg or uterus, commences cell identification and tissue growth. It takes nearly a month for a young chicken to hatch or nine months for birth in mankind. Development continues after birth, increasing cell number in specified tissues as growth sets in. I consider the need for protection in early animal development to be the need for certainty of limb number. Movement is as crucial for animals as light is for plants. If limb number were highly variable, coordinated movement would be impossible and food and mate acquisition impossible too. It is only after birth that movement commences. Movement of animals is the recognizable but familiar characteristic that is coordinated by a nervous system that operates at similar speeds. Together with musculature, this determines the relative constancy of speed of animal movement.

There is no doubt in my mind that it is failure to visibly see general plant movement that leads to misleading designations of plants as simple and unintelligent. The behavior of plants is developmental, while animal behavior is only limited by the speed of action potentials and muscles. Although both are called behavior, strictly speaking they are not comparable. Time-lapse photography of plant behaviors belies human assessments of plants as still life.^12^ (p. 13).

Animal complexity, if true, has its own hazards; more things to go wrong. The poor medical history of our species is strong support for this view. But the blame surely lies on an unbalanced evolutionary history; it's been rushed. Angiosperms appeared over 100 million years ago and have had plenty of time to sort out problems via evolutionary selection. The evolutionary history of *homo sapiens* is, at best, one twentieth of that and that is not long enough for significant selection to take its full toll. During that short period of evolution, the size of the brain has tripled along with a much heavier cranium and a poorly developed, S-shaped spine to support it when walking. It is an anatomical dysfunction known to cause very frequent medical problems. As for human intelligence which our species, *Homo sapiens,* places above all other organisms, past and present human history belies that assumption too. Wise we are not. Russell^14^ considers that *Homo faber* (the craftsman) is a better designation of mankind. Our technology is superb. As a species, we are good at making things and taking others apart to see how they work. The natural human bent is mechanistic and reductionist. The zoological perception that animals are more complex, the greater the fascination in uncovering how it works. That probably explains why most biologists research animals; excepting obvious medical value. Reductionism indicates the failure to think in systems terms.

### The commonality of signal transduction between plants and animals. Plants as green animals!

One common view, that can lead to botanical rejection, was to imply that plants were green animals. This was certainly the reaction to and the reason for rejection of plants as intelligent organisms.^15^ The thinking that led to the intelligent plant in Trewavas^2^ was fueled directly from experimental observations. Aequorin-transformed plants detected [Ca^2+^]_I_ transients lasting some 20 seconds. The lag period in [Ca^2+^]_I_ to a signal such as touch or brief wind stimulation was either a tenth of a second or less. How could something so fast lead in due course to something so slow as plant behavior? Eight different environmental signals generated transient kinetics in [Ca^2+^]_I_ unique for each signal and it was clear that this was learning about the environment.^16^ ^,^ ^17^ More significant were the observations that slight but selective inhibition of the [Ca^2+^]_I_ transient to three different environmental signals blocked the induced transcription of genes normally associated with the specific environmental signal.^18-20^ Changing gene expression can be slow and may account in part for the relative slowness of much plant behavior. But the phenomenon of priming results from altered gene expression and is now recognized as a prominent plant memory.^21^

Bhalia and Iyengar^22^ had described the emergent properties of the animal signal transduction network built around [Ca^2+^]_I_. This network is largely congruent to that known in plants. These authors concluded that signal transduction enables cells to learn about their environment and reported that an emergent property of this system was to construct a memory. This was referenced as an unusual form of intelligence,^17^ because learning and memory are the essential bases of all forms of intelligence. On that basis, intelligent behavior via signal transduction networks was applicable to plants too.

By 1991, cell signaling through [Ca^2+^]_I_ was already well-established in animal cells including nerve cells (e.g.^23-25^). Nagata et al.^26^ described 20 homologous genes involving calcium signaling between *Arabidopsis* and *Homo sapiens*. Many other homologous genes such as MAP kinases, protein kinases, phospholipase C, specific Ca^2+^channel proteins and others have now been detected in plants. One omission is an apparent absence of the GTP-binding protein, Ras, but there are other small GTP-binding proteins that may replace its function. The use of cyclic nucleotides in animal cells had been described by the middle 1950s and is now well-established as a signal transducer in plants too.^27^ Nucleotide cyclases commonly act as specific developmental and metabolic receptors in both animals and plants, including single-cell plants e.g. *Chlamydomonas.* ^28^ The molecules to be sensed, however, do differ substantially between plants and animals. The expectation is retention of an enzymatic locus but modification of the remaining protein sequence to construct the specific sensor array, which has been found to be the case Gehring and Turek.^29^

The divide between animal and plant eucaryotes is considered to have occurred at an amebic stage. Two billion years ago, an amebic ancestor engulfed a blue-green alga that with time evolved into the chloroplast via an intermediary symbiotic state.^30^ ^,^ ^31^ That amebic ancestor clearly used [Ca^2+^]_I_ as do present-day amoebae.^32^ ^,^ ^33^ *Paulinella* is an ameba containing a captured cyanobacterial chromatophore acquired about 100 million years ago and a clear indicator of the mechanism of the critical first step in plant evolution.^34^

Amoebae can associatively learn and that includes memory.^35^ Such capabilities must have passed into the putative plant ancestor because they are still present in plants. The first indication of a short-lived, cold-induced memory was described.^18^ Evidence for plant memories is now well established and described later. In this particular transduction and cellular context, describing plants as green animals is justified.

## Survival and adaptive are generic terms; together they underpin intelligence

The definition of intelligence used by Trewavas^2^ was constructed by Stenhouse^3^ for a zoological audience. There were objections raised to its use because plants are not animals.^15^ Objectors, however, failed to recognize the generic nature of the terminology used; adaptive, probability of survival, lifetime and individual refer to all living organisms and the “Individual” is characterized by Darwinian selection.

### The driving force that underpins life

Life has a driving force built in; a fundamental property that underpins survival to enable reproduction.^36^ The drive to survive was what enabled the evolution of life in the first place. It differs in intensity between individuals because of individuality, the molecular and genetic noise that results in individual variation.^37^ ^,^ ^38^ Two examples exemplify the power of this drive to survive. Living bacteria can be found thousands of feet down in rocks^39^; tardigrades resist freezing temperatures and the vacuum of space^40^ amongst others. No life can exist without this fundamental capability.

Investigation dates the origin of life to about four billion years ago, not long after the earth itself was formed. Present evidence is based on identification of potential fossils in rock sections, 3.8 billion years old, accompanied by isotope discrimination between C13 and C12.^41^ Stromatolites are clearly present by 3.5 billion years. Lepot^42^ has illustrated some potential and unusual fossil structures of very early life. Kanaparthi et al.^43^ suggest these look similar to lysed L-type bacteria. Life may have evolved without a wall and thus in low salinity environments.

Dill and Agozzino^44^ identify the driving forces that underpinned life's origin. “We discuss four major prebiotic ‘discoveries’: persistent sampling of chemical reaction space; sequence-encodable foldable catalysts; assembly of functional pathways; and encapsulation and heritability. We describe how a ‘proteins first’ world gives plausible mechanisms”. The drive to survive and reproduce is basic to all life. Without that drive, there is no continuation of life.

Up to the present day, substantive environmental shifts have occurred resulting from continental drift, volcanic activity to variable extents, and damage by impacting extra-planetary debris. Douglass^45^ illustrates a temperature history of Earth indicating that it has varied by at least 30 °C between epochs and has seen six cooling periods with five of them in the last two billion years including snowball earth. The origin of plants is placed at around 1.8–2 billion years ago.^31^ Despite these severe environmental challenges, life continues because the earliest forms were built around mechanisms that confront and counteract the threats that abound. Evolutionary processes favor traits and behaviors that enhance individual survival and contribute to the potential maintenance of the species. Such behavior is also evident in our own species; the need for safety, food (energy), shelter, and reproduction often leads to a search for purpose and social connection that have direct biological origins. Adaptive behaviors (traits) increase the probability of survival of the individual during evolutionary challenge^1^ but do not guarantee it.

Intelligence is “behavior that is adaptively variable during the lifetime of the individual” and I indicated it for adaptive behaviors in plants.^2^ Survival and adaptive are generic terms. They apply to all present-day living organisms (plants, bacteria, protozoa, animals) that experience challenges to individual survival in wild circumstances and use adaptive behavior to increase the probability of survival. Biologists need to reject the one-species, human, veneer placed on intelligence and instead assess intelligence biologically. Life itself is intelligent and survived only because it evolved adaptive mechanisms. Earth is a planet dominated by plants; that is a good measure of evolutionary success and intelligent capability.

### How did Stenhouse arrive at his definition?

Stenhouse's book was published in 1974. The animal literature on behavior in 1974 tended to center around instinctual behavior, an heritable, apparently rigid behavioral pattern, sometimes assumed encoded in the genes and claimed to be a genetic blueprint. But instinct was still regarded as adaptive within the normal circumstances of the organism in question. Even^46^, (p. 237) saw the fallacy of these claims about the rigidity of instinctual behavior. The behavior of protozoa was commonly thought to be rigidly defined. But as Jennings^46^ states “These ‘definable’ reaction forms are usually within themselves variable within wide limits.” Internal conditions and environmental changes are of the greatest importance in determining behavior. More recently, Lehrman's^47^ withering criticism of instinct as proposed by Lorenz and Tinbergen emphasized instead the role of learning and development in these behaviors. Blumberg^48^ provides an excellent critique of instinct concluding that they are not satisfactorily described as inborn, preprogrammed, hardwired or genetically determined; they are learnt during development. In *Homo sapiens* the only “instinctual responses” are the eye blink and the knee jerk which result from a reflex arc. Thus, Stenhouse arrived at behavior that was variable and adaptive, leading to improved survival during evolution that formed the books title.

The equivalent “instinct” in vascular plants might be the developmental cycle; seed to flower to seed. That seems naturally invariable in gymnosperms and angiosperms. A further equivalent “instinct” in plants might be the response to gravity. Virtually all plants are gravi-sensitive. Ma and Hasenstein^49^ demonstrated that acquisition of seedling gravi-competency was determined by the position of the seed with respect to the gravity vector. If the embryo root pointed vertically upwards, only 25% of seed roots were able, in due course, to assume a downward growth direction. The most divergent form of behavior from supposed instinct is of course non-rigid, non-fixed; that is, variable behavior and the category that has most meaning for plants.

## Plant intelligence is easily defined by the dictionary definition of intelligence

The Collins English dictionary (5th edition^50^) states that intelligence is the ability to perceive and understand the “meaning” of that perception. Plants perceive enormous amounts of environmental information because they do respond positively to them. A list of environmental parameters that are sensed has been tabulated under ten different categories.^12^ (p. 70). Perception of that information leads to physiological and phenotypic changes, many of which are clearly adaptive and can improve the probability of survival.

Kull^51^ has provided a valuable article on the concept of biological “meaning.” He identifies the commonest biological use equates “meaning” to functional or purposeful correspondence. Since purpose is considered later, it is meaning as biological function correspondence that is considered here.

A second common use equates meaning to survival or evolutionary experience.

Using inbred lines of *Polygonum cespitosum*, Sonia Sultan^52^ provided photographic examples of seedlings grown firstly, in dry (droughted) soil; secondly, in effective shade. The phenotypic changes were compared to controls experiencing optimal conditions for growth (These two photographs can also be found in Gilroy and Trewavas).^53^

In drought conditions, the *Polygonum* root system was massively increased. The lack of water has been perceived and the meaning of that perception has led to massive root proliferation. A prime function of the root is to provide the plant with water. This is therefore an adaptive response and thus intelligent by the previous definition and that of the dictionary. The shoot is also massively reduced with probably increased cuticular thickness covering the few leaves. Premature flowering may provide seed that could, via transgenerational inheritance, be better adapted to the water-poor environmental conditions. Or they could remain dormant and only germinate when conditions improve.

There are correlative changes which result from the massive sequestration of carbohydrate for root proliferation. The lack of water is perceived first by the root meristems or early elongating root region.^54^ Continuation of drought conditions leads to massive reduction of leaf synthesis with a thicker cuticle. These adaptive responses reflect species occupation of areas with uncertain or highly variable rainfall and involve eventual adaptive responses by the whole organism.

The second treatment was to place *Polygonum* plants in effective shade, thus severely limiting available light energy for photosynthesis. A primary function of leaves is to photosynthesize and provide the plant with carbohydrate resources. The reduced light intensity was perceived by the leaves (and possibly the stem) and transmitted to the shoot meristem. The meaning of that perception has been understood by the synthesis of leaves with greatly increased leaf area, thus compensating, in part, for the light-poor environment. Usually, such leaves are thinner with a reduction in the number of mesophyll layers. This intelligent adaptive response is to gather as much light as is possible in this light-poor environment. Again, these are adaptive responses.

The shade avoidance syndrome is an alternative but intelligent response to perceived shade that leads to an elongation of internodes with reduced thickness. The change in elongation rate might enable the individual plant to reach more satisfactory light regimes and eventually achieve greater seed number via greater flower exposure. There are simple relationships between gathered and stored resources and the number of seeds that can be provisioned.

In the two examples described, lack of water or light, the adaptive changes reflect attempts to overcome the environmental obstacles that hinder survival of the individual and, in turn, the species. Plants, by perceiving the resource imbalance and understanding the meaning of that perception by proliferation of roots or thinning of leaves, fulfill the dictionary definition of intelligence.

Dictionaries also contain intelligence definitions such as good mental capacity. Such qualities are limited, by communication, to one species, mankind. Because of our inability to communicate meaningfully with virtually all other species on earth, whether the other eight or more living species on earth have good mental capacities remains, unfortunately, entirely speculative.

## Artificial intelligence and biological adaptive intelligence

The AI and computational expert,^55^ (p. 1–19) provides a valuable but critical discussion on the early failings of Artificial intelligence (AI) as it relates to the nature of intelligence; criticizing in particular the narrow context in which early problems of intelligence were to be solved. He argues (Beer.10) that intelligence is the much broader ability to cope with the complex, dynamic unpredictable world which all life (including mankind) experiences. “Our higher cognitive functions are our own particular human elaboration of this more basic competence for effectively coping with the world”^55^ (p. 11). The more “basic competence” reflects evolutionary strategies and adaptive capabilities^3^ (p. 31) of all animals, plants, and microbes in natural environments. His statement mirrors earlier statements by the prominent psychologist, Anastasi,^8^ who recognized that intelligence is adaptive insofar as it represents ways of meeting the demands of a changing environment. Such behavior varies with the species and the specific environments in which it lives. Again, this emphasizes the generic nature of intelligence and adaptive behavior.

There is of course, great interest in artificial intelligence, but the cell and the computer are distinctly different entities. Although there is some form of structural arrangement through membranes and organelles, the cytoplasm itself still acts as a semi-fluid structure, and the cell operates as an open system. Protein complements are changed as does their interactions. Computers, no matter how complex, still have structural defined connections and thus cannot directly mimic the nature of cells. Nobel and Noble^56^ continue some useful discussion on these issues.

George Romanes was a close colleague of Charles Darwin during the last eight years of Darwin's life. The interaction is described briefly in Trewavas.^57^ He wrote the first book on Animal Intelligence (1883). Romanes defined biological intelligence^58^ (p. 17) as “intelligence is the faculty which is concerned in the intentional adaptation of means to ends”. The “means” references the behavioral capabilities of the organism and the “ends” refers to evolutionary benefit. I interpret his intentional adaptability as meaning adaptive. His close association with Darwin enabled him to correct Darwin's (naïve) view of intelligence. Darwin thought that if organisms behaved as he would, then that behavior is intelligent. Romanes pointed out to Darwin that there had to be profit (improved survival) in the behavior, or it was not intelligent.

## Psychological abandonment of IQ as a measure of human intelligence in favor of adaptive behavior

Alfred Binet was a French psychologist who authored a short text on protozoan behavior and concluded that such organisms were intelligent.^59^ In the early 20th century, the French government asked Alfred Binet and Theodore Simon in 1905 to construct a series of tests to help identify children with special educational needs. The original test in French quickly attracted the interest of other psychologists because they felt it more accurately assessed the higher and more complex functions of the human brain. These psychological measurements blossomed as other psychologists realized they could investigate various aspects of mental ability and thinking, such as Spearman's “g” (general mental ability) suggested in 1904 as a single underlying ability for all cognitive tasks.

Most plant scientists will be aware of some aspect of those terms identified as IQ, (intelligence quotient) since they have become the public view of intelligence. Anastasi^8^ summarized IQ as simply measuring scholastic aptitude. That description, which I consider correct, indicates its limitations.

The leading psychologist, Robert Sternberg, is the most prominent contributor to the enormous psychological literature on human intelligence. He has published over a thousand articles, chapters in books, and authored and edited numerous books on the subject of human intelligence. He developed his own triarchic theory of human intelligence, involving analytical, creative, and practical aspects of human intelligence. His cognitive psychology is in its fourth edition.^60^

Despite his long history on intelligence as IQ and g (Spearman's general ability), he now firmly rejects them, arguing that human intelligence is instead adaptive behavior.^7^ ^,^ ^61-64^ “Adaptive intelligence-intelligence that is used in order to serve the purpose of biological adaptation in real world contexts”.^61^ “Without adaptation, one has little or nothing resembling intelligence”. “Our latest work on adaptive competence is based on my theory of adaptive intelligence, and always has been primarily about adaptation to the environment”. The basic idea of the theory is that we can no longer afford to define intelligence merely as [Spearman's] g or IQ. Doing so has been a disaster-literally, not merely figuratively. At the same time that IQs have “benefited” from the 30-point increase of the 20th century, the world has been coping—or often failing adequately to cope—with disasters that are perhaps unprecedented in human history. These disasters include human-induced global warming, air and water pollution that are killing millions of people, income disparity that has resulted in staggering gains for the wealthiest 1% of the population at the expense of everyone else. “(https://robertjsternberg.com/successful-intelligence)”. “Theories of intelligence have failed to account for why so many people who have relatively high levels of intelligence as an ability, fail to deploy their ability especially towards positive ends.” ^65^ The positive ends are those that increase our species survival; the biological role of adaptive behavior.

Intelligence is an aspect of adaptive behavior, even in humans^66^ (p. 1). Artificial intelligence, human intelligence, animal and plant intelligence all identify the critical adaptive capability that over aeons of time has enabled life to survive. Adaptive behavior is an irreducible property of life itself.

## Cell signaling as a cognitive process

In the context of signal transduction, the word “cognitive” equates to “any relation established by interpretation.”^51^ What it concerns is the unique, subjective, sensory worlds inhabited by different organisms and, in this context, plant environments in wilderness. “Cell signaling as a cognitive process” is the title of a review by Koseska and Bastiaens.^67^ These authors consider that the function of a tissue emerges through intracellular networks that are shared between tissue cells. In this context, Koseska and Bastiaens are using the word cognitive to refer to the way in which knowledge of the environment is acquired. “On this basis, cell identity is not intrinsically predetermined by the genetic code but dynamically maintained in a cognitive manner”. For a non-neural version of cognitive signaling, see Stewart.^68^ In my view, this concept is an important advance in understanding the processes of signal transduction and communication. An associated topic is phytosemiotics. That is how plants interpret environmental information and use that information to understand its meaning. Previously in this article, it was indicated that plants interpret lack of water by specific proliferation off the root system and shade conditions by increasing the size of leaves.

### The problems of noise in signal transduction

It is not uncommon to find diagrams of plant signal transduction events in which the known constituents are placed in a box to indicate a cell. Arrows are then drawn between the transduction constituents indicating potential connections. This diagram is a useful summary, but it can also suggest that signal transduction is a simple process. The primary problem with the diagram is that multi-omics investigations have identified the quantitative variations in transcriptomes and proteomes between individual tissue cells. This is commonly referred to as cellular noise.^69-71^ The processes of signal transduction leading from [Ca^2+^]_I_ or cyclic nucleotides involve at least several hundred proteins.

Cellular noise indicates that there are likely to be substantial molecular variations in some or many of these proteins between individual cells. On a cellular basis, individual protein species in a tissue can be summarized as a Gaussian distribution.^71^ Transduction involves interactions between the constituent proteins. With variation in protein number, the simple rate kinetics of these interactions will also exhibit cell variation when transduction is initiated. This problem of evident variation is well exposed by.^72^ Provision of supposedly exact growth room conditions and laboratory operations, identical seed origin and soil used, still failed to get reproducible results on cell numbers and growth characteristics between ten different laboratories. Transfer that information to wilderness conditions and the real problems are laid bare. Physiology tries to obviate the problem of variation by the use of statistics, sometimes implying that the mean value is the only one worth considering. Variation is the reality of life; anything that fails to emphasize that fact entirely misleads.

### How then do cells manage with such variation?

Explanations involve networks of varying complexity and using feedback mechanisms which help but do not eliminate the variation.^70^ Noise applies to all cellular processes; not just signal transduction. The common result is individuality.^37^ Individuality is the substance of selection. Plants in wilderness are metapopulations of leaves, stems and roots whose number varies widely between individual plants. As indicated by Herrera,^73^ such variation enables plants to much more finely exploit the local resources than the animal with fixed numbers of limbs. Chapter 1 in Williams^38^ is a valuable introduction to the problems of variation and its consequences, particularly if students are told to find equivalent examples from the plant and fungal kingdoms.

### Do plasmodesmata control cognitive changes in plant cell signal transduction?

Crawford and Zambryski^74^ bombarded leaves with microprojectiles containing transgenic GFP. They were thus able to assess functioning plasmodesmatal connections from the observed movement of GFP into adjacent cells. They reported considerable aperture variation. Some plasmodesmata were fully open, some fully closed, and a range of aperture openings in between these two extremes. Age and physiological conditions were two features that additionally modified the active aperture numbers. Plasmodesmata exist in varying states and cell-to-cell connectivity is heterogenous.^75^ Molecular noise may be responsible and has important consequences for understanding tissue functioning.

### So what can be exchanged between plant cells during transduction that might help construct cognitive interpretation?

Tee and Faulkner^75^ provide a table that indicates that proteins about the size of GFP can move with relative ease between plasmodesmata if the aperture is open. Small GTP-binding proteins (GTPase), calmodulin, for example, would then likely equilibrate between adjacent cells with an open aperture. Calmodulin, when combined with its four Ca^2+^ ions binds to and activates several hundred proteins.^76^ sRNAs and peptides should also equilibrate by diffusion between relevant cells. But a critical question surrounds the initiating molecules that are generated as a result of environmental change, [Ca^2+^]_I_ , cyclic nucleotides, GTP-binding proteins, etc. Would any be shared as required for cognitive signaling?

The apparent consensus is that elevation of [Ca^2+^]_I_ blocks plasmodesmatal transmission.^75^ ^,^ ^77-80^ Concentrations of [Ca^2+^]_I_ higher than 1 µM can activate gelsolin and villin that lead to fragmentation of actin cables, essential to plasmodesmatal closure control.^81-83^ Callose synthase is activated slightly beyond 1 µM Ca^2+^ and blocks plasmodesmatal aperture.^84^ Fortuitious closure can result if [Ca^2+^]_I_ exceeds this critical concentration. Plasmodesmata contain calcium-binding proteins also thought to be responsible for initiating closure.^80^

### Imaging the apparent movement of the calcium signal across leaf tissues initiated by a localized cold shock source

When single plants containing active aequorin are placed in ice-cold water, they generate immediate [Ca^2+^]_i_ transients that reach a peak 1–1.2 µM [Ca^2+^]_I_ from a resting level of 0.05–0.1 µ M in less than 0.1 second. The resting level is recovered in about 15 seconds.^20^ We have imaged some of these Ca^2+^ transients in leaves and roots.^85^, ^86^ A cold shock stimulus was applied at a discrete but tiny part of the leaf. After a brief lag, a divided but elevated [Ca^2+^]_I_ signal was recorded that progressively moved away from the inducing source and across the leaf but disappeared within 2–3 minutes. Providing a cold shock to the roots leads after a lag of about three minutes to an elevation of [Ca^2+^]_i_ in the leaves.

Pavement cells respond to touch of a single cell by inducing the formation and directional movement of calcium waves.^87^ The time course of the Ca^2+^ movement in pavement cells is very similar to Knight et al.^86^ taking several minutes to traverse the leaf from a point of impact before fading away. Transmission of insect damage induces a Ca^2+^ wave dependent on glutamate synthesis and glutamate-dependent channels.^88^ It would be useful to examine whether cyclic nucleotides likewise move between cells when synthesized is elevated.

Holdaway-Clarke et al.^77^ have clarified some of the uncertainty over elevation of [Ca^2+^]_i_ and aperture control. If the elevation of [Ca^2+^]_i_ in the plasmodesmata is in the physiological range (below 1 µM), then plasmodesmatal closure lasts only about 10–15 seconds. By iontophoretically loading Ca^2+^ into a plasmodesmata, closure is limited to some 10–15 seconds. These authors suggest that the ER in the plasmodesmata may sequester physiological levels rapidly, enabling rapid recovery of opening after transient closure.

What then could interpret the apparent movement of elevated [Ca^2+^]_I_? The plasma membrane of cells is contiguous with the connecting plasmodesmatal plasma membranes. In turn, this is contiguous with that of the plasma membranes of the adjacent cell. Initiation of a Ca^2+^ transient in one cell is then shared through all the connecting plasmodesmatal plasma membranes and thence to adjacent connecting cells.

### Wherein lies the significance and indication of cognitive meaning?

In animal cells that have gap junctions, elevation of [Ca^2+^]_I_ in one cell seems to be shared to just a few others in the form of a simple wave.^89^ ^,^ ^90^ In plant leaves, anywhere from 5 to 25% is vascular tissue. Bose^91^ demonstrated that the phloem transmits electrical signals. These electrical changes due to cold and touch will surely impact the phloem during their progress and initiate electrical information, which can then be transferred towards the apical meristem. Light/dark variation is a primary signal altering the electrical potential characteristics of leaves.^92^ Conveyance of that electrical information to the apical meristem could be the signal that results in the shade avoidance-adaptive mechanisms of increased length and diminished thickness of stem tissues. Other recent information has reported that leaves touching each other increase the stress potential of the participants.^93^ ^,^ ^94^ Cold signals have obvious preparative effects, including abscission via the pulvinus. These are all cognitive events using signal transduction.

## Biological purposeful behavior. The directiveness of organic activities towards goals

“The Directiveness of Organic Activities” (1945) is the title of a small book by the far-sighted biologist, E.S. Russell^14^. He used the term “directiveness” in preference to purpose. In 1945, the ‘Modern Synthesis of Evolution’ dominated evolutionary thinking rather dogmatically claimed that life was purposeless; evolutionary variation was random. Any claimed evolutionary direction was a misinterpretation; it was all down to chance. And like all such embracing ideas, it rapidly became dogmatic. For example, “The universe we observe has precisely the properties we should expect if there is, at bottom, no design, no purpose, no evil, no good, nothing but blind pitiless indifference”^95^ (p. 133). Russell's groundbreaking book has been consequently ignored.

Biological purpose, that is, goal directiveness, is easily demonstrated in plants. A flowering individual can be generated from a single somatic cell; a single protoplast; regeneration from callus; regeneration via stem, root and leaf cuttings; regeneration from damaged embryos and from individual plants damaged to varying extents by disease; via stiff competition, herbivory or from mixtures of these and of course finally from the growing seed. Some plants like *Kalanchoe* simply construct plantlets that drop from leaves and become individuals in their own right.^96^ There are also some 50 environmental variations known to affect plants and species and from which they survive and then flower^12^ (p. 70).

While the routes are multiple, the single goal, the flowering, individual, can be reached from any of the above starting points. Phenomena like these are the essence of biological purpose. Purposeful behavior has the characteristics of feedback control, but it is unlike the simple feedback controls that are familiar, such as end product inhibition of the first step of a metabolic pathway. Development itself can be accessed from many different starting positions!.

The word teleonomy has been coined to indicate biological goal-directed behavior and replaces teleology. The Nobel prize winner^97^ (p. 20) makes it clear. “*One of the most fundamental characteristics common to all living organisms without exception; that of being objects endowed with a purpose or project. Rather than reject this idea as certain biologists have tried to do, it must be recognized as essential to the very definition of living beings. We shall maintain that the latter are distinct from all other structures or systems present in the universe by this characteristic which we shall call teleonomy*”. Goal-directed behavior is a basic characteristic of life itself. There are some interesting consequences that follow. Like survival, reproduction of some kind has always been essential to the continuation of life itself. Survival and reproduction are thus irreducible properties of life. Biological purpose is quite simply goal-directed behavior. There is no implication of conscious control in biological purpose (Vane-Wright and Corning^98^).

A simple change in language indicates purposeful behavior. It is correct to say that a seed germinates and makes a new plant. To say instead that a *seed germinates to make a new plant identifies* the purposeful nature of germination. A flower opens to attract pollinators, a fruit ripens to attract animal consumers that distribute its seed; leaves open to increase light absorption; stomata close to reduce water loss; a root branches to increase access to minerals and water for uptake, etc.

## Plants in wilderness are purposeful agents

Plants exhibit agency in wild circumstances; a capability exemplified by the ability of a single plant to control its own responses to environmental challenge in a purposeful, goal-directed manner. Plants are not passive organisms; they assess their present state and merge this information together with the perceived environment, their previous and present history and make judgements over the present and potentially future threats in order to accomplish survival and eventual reproduction. Agency upsets a common view of plants as passive organisms at the mercy of any environmental shift.^99^

In the 19th century, Julius von Sachs was well aware that plant behavior was purposeful. “*All those adaptations in the organism are purposeful which contribute to its maintenance and to its existence* ^100^(p. 601). “*Concerning one point I should wish to anticipate: viz, the use of the word ‘Purpose’, a word which many fanatics of the theory of descent would if possible banish entirely from the language*”^100^ (p. 10). “*By the expression this-or- that mechanism has a purpose in an organism one understands nothing more than this contributes to the ability of the organism to exist*”^100^ (p. 10).

The Modern Synthesis of evolution^101^ assumed randomness in mutation followed by selection. Dawkin's^102^ “selfish gene” concept best illustrates this issue. Purpose was rejected on the basis that it implied potential evolutionary direction. Lamarckian inheritance was rejected because it implied that the environment could directly modify the genome. Finally, the Modern Synthesis was claimed to be Darwin's view. Ideas on evolutionary mechanisms are changing. What Lamarck actually said is listed^12^ (p. 211–219). Lamarck raised the issue of domestication and the multiple end points obtained by selected breeding used by Darwin.^36^ He indicated how plants vary enormously in structure when grown in different environments.

Evidence summarized below indicates that change is required, particularly in our understanding of the mechanisms that result in individual variation and thus eventual selection. They do not negate the structure of evolutionary change as it has happened in the past. Kutschera^103^ provides a valuable recent article describing the process of evolutionary change since life started in animals and plants.

### Understanding of evolutionary mechanisms is changing

(1) *Darwin's views on selection had emphasized equally the importance of the environmental conditions of life alongside competitive selection.* ^53^

“In my opinion the greatest error which I have committed has not been allowing sufficient weight to the direct action of the environment”.^104^ In Darwin^105^ under the heading of “Direct and definite action of the external conditions of life” Darwin summarizes some of the evidence that concerns his “error” referred to above. I suspect that a goodly number of Darwin's observations (1868) are due to transgenerational inheritance that is common in plants.^53^ Darwin's appreciation of environmental influences are indicated in Darwin^105^ and the following chapters.

(2) *Mutation frequency in the genome is not random. Cells decide what to repair.*

The supposed randomness of genome mutation was not based on actual evidence but supposition stemming from X-radiation damage to the genome in the 1920s. Recent research has shown that mutations in the genomes of bacteria, fungi, animal and plant are not randomly distributed. Cells use DNA repair and target repair to certain critical genes and those frequently transcribed.^106-109^ The cell overrides mutation randomness; it's not left to chance.^56^ ^,^ ^110-112^ The book by^110^ describes where the current understanding of life is incomplete or often wrong.

(3) *Cells manipulate their own genomes. Natural genetic engineering.*

Increasing knowledge of genome structures and sequences, starting with Barbara McClintock's studies, has generated the concept of natural genetic engineering: cells manipulating their overall genome structure.^113^ ^,^ ^114^

(4) *The basic unit in evolutionary change is the cell which is alive; not the gene.* ^110^

A molecule of DNA contains enormous numbers of base sequences coding for genes. Their meaning is only apparent inside the cell.

(5) *The selfish gene is a misnomer.*

Genes are often regarded as central to plant biology but only because they can be modified and act as memory. The richness and meaning in protein–protein interactions and their structures is far greater than speculation on the role of genes.^115^

(6) *Lamarckian inheritance is common in plants.*

Jablonka and Raz^116^ describe the critical relevance of transgenerational inheritance via epigenetic modification of the genome as indicating Lamarckian, not Darwinian, inheritance.^116^ Transgenerational inheritance can last anywhere from 1 to 9 generations.^53^ ^,^ ^117^

(7) *The Weismann barrier.*

A supposed barrier to separate reproductive from somatic cells. No such barrier exists in plants. The cells that construct the vegetative meristem are usually the parents of those that construct the reproductive meristem. Other modern synthesis failings are described in Noble.^112^

(8) *The necessity to incorporate teleonomy (teleology) into evolutionary explanations.*

“Giving organisms purposes and goals is a profoundly adaptive strategy, for it equips them to act as agents” Ball.^110^ Teleonomy evolved because it increased reproductive fitness.^118^ ^,^ ^119^

### What new theory of evolution better fits the facts

Corning^98^ has proposed a theory of evolution incorporating purpose. However, the evidence provided for this theory derives predominantly from animal examples. Purposive goal-directed interactions within individual plants and between plant species and non-species members are so endemic that any new construction of a theory of evolution has to include purposive behavior of plants as a strong contributory element to individual species survival.

The kind of evidence that could be included for a general theory of goal-directed processes could be convergent evolution in plants. Fifteen examples are published in Trewavas^12^ and more are to be found in Gilroy and Trewavas.^53^ Transgenerational inheritance; that is, the purposeful provision to siblings of parental experience who live within local parental environments. Diecious species; male flowers only in N-poor environments. The genotype via pollen moves from the local poor area. Female flowers are synthesized in N-rich environments, areas that are good for seed growth and subsequent siblings.^12^ p. 184. The ubiquity of leaf-like structures in all phylae.^53^ Cambial appearance and development, the goal being to increase trunk size, to demarcate resources to advantageously placed shoots^12^ p. 105 and to outgrow competitors. Reaction wood supports larger branches. In damaged stems, cambial interruption regenerates the ring structure^12^ p. 110. Bisecting apical meristems leads to regeneration of two new meristems. Numerous adaptive behaviors improving the probability of survival. As for evolutionary direction, plant form and function are constrained by physical laws that could provide some semblance of overall evolutionary direction.^53^ Those that don't obey the physical principles don't survive. These suggestions are some obvious examples. Each represents purposive behavior. The hypothesis needs a plant review in its own right.

## Tropic phenomena are goal-directed and purposeful in their execution. Two sensory systems and transduction routes in gravitropism

According to Pfeffer,^120^ there at least a dozen tropic phenomena. If any or all of these are adaptive, then fail-safe mechanisms of some kind should exist to deal with anomalous environmental variation and the potential problems of cellular noise. Out of these, responses to gravity and light corner most research.

I have chosen gravitropism because sufficient evidence indicates that there are two gravi-sensory systems in plants. Textbooks often provide a simple diagram of gravitropism using statoliths. In my many years of teaching, such simple models, and the adherence to them, create the view amongst biological students that plants are indeed simple organisms; nothing of interest worth further study^120^ (p. 211) states that sensitive roots and seedling stems perform slight curvatures in response to a centrifugal force equivalent to 0.001 g. The sensitivity indicated by this measurement, in itself, raises issues about mechanisms, particularly whether statoliths could sense such tiny stimuli.

### What situation would require a second (fail-safe) mechanism?

The current mechanism of gravitropic responses identifies starch statoliths as the sensing component and currently it is well-established. But is starch always available? Thalmann and Santelia^121^ have reviewed the conditions under which starch is depleted and it is, unsurprisingly, stressful conditions. These are conditions likely to be frequently experienced in wilderness. And even if some starch is unavailable, Kiss^122^ has argued that full statolith size is necessary for full gravitropic sensitivity. Inadequate starch content is then likely to interfere with the anticipated alignment to the gravity vector, impacting on the probability of survival and diminishing adaptive behavior. Is the present model of gravitropic behavior lacking an essential fail-safe alternative?

### Electrical changes and mechano-transduction suggest an alternative sensory system

Using aequorin-transformed *Arabidopsis* seedlings and rotating them on a wheel initiates a rapid calcium transient lasting less than a minute but followed by a much slower calcium transient peaking after several minutes and followed by a decline lasting some 30 minutes or more (Plieth and Trewavas).^123^ Many publications report that placing a vertically growing tissue on its side initiates rapid electrical changes often within 30 seconds to one minute).^124-134^ These electrical changes can last for 25–30 minutes or longer^124-126^ and occur both in the shoot tip and the zone of expansion.

When laid flat, the lower side becomes more positively charged than the upper, thus altering the internal electrical field structure.^126^ Becoming more positively charged can be considered to result from the increased entry of wall calcium into the cells. Reducing the establishment of an electrical gradient between the two sides of a tissue gradient by shunting the external electrical potential reduces gravitropic bending by a half.^131^ Gravi-stimulation of intact *Arabidopsis* roots also initiates a calcium wave moving from the tip to the growing region similar to auxin movement^135^ but again just on the lower side. Initiation and propagation of variation potentials may be a possible further consequence.^136^

Lipid rafts can move around the plasma membrane, dictated by the shape and structure of the cellular electrical field.^137^ PIN 2 must be a moveable protein to accommodate auxin movement for different spatial directions during tropic bending.^138^ It can be suggested that PIN2 is simply located in a lipid raft. As the relevant ionic field changes, it will move around the plasma membrane to the appropriate position to divert the auxin stream and alter growth, as indicated in the standard auxin-based theory of gravitropism.

### Additional gravity-induced observations need explanation

Firn and Digby^139^ (p. 135) reference eight papers that show removal of the tissue tip (the statocyte region), left stumps that were still gravi-sensitive. These authors also reported that the outer epidermal layers constrain normal growth. Caspar and Pickard^140^; Pickard and Thimann^141^ reported that “starchless” plants still express a strong gravitropic behavior in seeds, roots, flower stalks and hypocotyls. Edelman^142^ stripped the vascular tissue and associated statocytes from cereal coleoptiles and in separate plants, removed the root tip. Both manipulated tissues still exhibited a strong gravitropic response. Single-cell plants respond meaningfully to gravitational change and direction^143^; Roux et al.^144^; Salmi et al.^145^.

### A suggested second signaling system (i). Mechanotransduction

If a vertically growing epicotyl or hypocotyl is laid flat on a surface, the cells on the lower side experience compression from the weight of the tissue above; those on the upper side experience tension. If instead the tissue is held at one end, a frequent observation is that the tissue bends as a result of its weight.^124^ ^,^ ^125^ Again, greater compression on the lower surface, tension on the upper. Compression and tension are mechanical signals. The act of laying a vertically growing tissue in a horizontal position constructs a mechanical gradient between the lower and upper side. Individual cells will experience this gradient with the maximal difference between the epidermal layers on the top and bottom.

Mechanical signals are interpreted via mechano-controlled calcium channels.^146^ The expectation is a gradient of [Ca^2+^]_I_ signaling from the lower to the upper side. Developing and growing plant cells in these tissues experience these mechanical forces through the viscoelastic structure of the cytoplasm. The energy available from this simple mechanical signal is estimated at 1 dyne/cm. Root and shoot cells respond in opposite ways to tension and compression.

An analogous stimulus is a single touch on one side of a vertically growing tissue. When this is performed, the side which is touched experiences compression and the untouched side tension. Bose^124^ (p. 437) illustrates a simple device for permanently touching a stem and showed that touch induces a curvature kinetically similar to that of gravity. Single transient touch signals also initiate a [Ca^2+^]_i_ transient, lasting about 20 seconds,^147^ or calcium waves.^87^

## Transduction of mechanical signals emphasizes possible transient changes in the nuclear position and resultant transcription change. The actin refit model

The nucleus is the largest and heaviest organelle of eucaryotic cells. During cell development the nucleus is specifically positioned, suspended, and held in place by actin-based cables attached to the plasma membrane and microtubules that are sensitive to control by [Ca^2+^]_i_.^148-151^ Mechanical forces on cells are transmitted into the cell via the conductive and viscoelastic cytoskeleton and impact directly on the nucleus itself.^149^ ^,^ ^151-153^

Just as there is a gradient in compression/tension within a single tissue, there will also be a gradient in Ca^2+^ entry between the upper and lower plasma membranes of the cells in these tissues. When a cell is experiencing compression, [Ca^2+^]_I_ entry likely disaggregates some local actin cables, probably through the Ca^2+^sensitive gelsolin. This will permit slight downward movement of the nucleus or even slight deformation of nuclear structure.^148^,^152^, ^154^ The extent of nuclear movement in different cells of a tissue laid flat will reflect its position in the compression/tension gradient. The cytoskeleton will experience the compression change too. Entry of Ca^2+^ into the cell via the plasma membrane is known to cause the local accumulation of the highly negatively charged PIP2 on the inner plasma membrane.^155^ Aggregated PIP2 in turn attracts proteins that act as actin nucleation sites,^156-159^ enabling new actin cables to regrow and encompass the nucleus. This is called an actin refit.

Via Lamins in the nuclear envelope, these new cables penetrate the nuclear envelope and then attach to the nuclear actin, whose known function is chromatin remodeling.^160^ Recovery and resetting of these new actin cables resets the nuclear position. Contraction of these new actin cables acts via nuclear actin to expose new chromatin regions for transcription. Specific wall loosening enzymes (e.g. plasma membrane H^+^ ATPase) are synthesized. The compression gradient will therefore be mirrored by a wall extension gradient causing the tissue to bend.

This hypothesis offers a route for investigation and testing its validity being based in part on what is established and now needs further examination.

The above mechanism, using changes in [Ca^2+^]_I_ and nuclear repositioning, indicates how plants without statoliths can still function gravitropically. It may also be the mechanism that enables single plant cells to respond to gravitational signals. Salmi et al.^145^ observed a discrete change in calcium current as the single cell responded to a gravity change. This mechano-transductive, actin refit, alternative can now help explain results that previously lacked explanation.

## Learning and memory

Learning and memory are the bases of all forms of intelligent behavior: artificial intelligence; immune intelligence; swarm intelligence; human intelligence and biological intelligence. A memory cannot be acquired unless it is first learnt. Memory is a direct indication that learning has taken place. This requirement for learning rules out the kinds of behavior which in animals were formerly regarded as instinct. When environmental conditions interrupt or threaten the completion of that life cycle, adaptive behaviors are invoked, enabling the identification of plant behavior as intelligent.^2^

### Learning

Barron et al.^161^ have collected 58 different definitions of learning and presented a composite definition. “A common umbrella concept that can be applied across disciplines by considering learning simply as the processing of information derived from experience to update system properties”…… “That will facilitate the integration of evolutionary approaches to learning.”

This general definition is designed to highlight the fundamental requirements of any organism that lives and learns in variable and uncertain circumstances that both provide its sources of energy and contain threats that can eliminate the individual. That fundamental process is the acquisition and processing of environmental information that, when interpreted (to update the organism)can enable avoidance of threat, construction of resistance, or perception of mates. Learning is fundamental for all organisms like plants that live in the wilderness. Life is about survival, as indicated earlier in this article. Nor does the definition specify the precise mechanisms used by different members of the 10 million or so species on this planet because that would defeat its intention of indicating what exactly is fundamental about the learning process.

### The first stages of learning. Potential complex transduction structures on the plasma membrane involving Ca^2+^

Lipid rafts offer some very specific possibilities for the first structural steps of learning through [Ca^2+^]_I_.^162^ Virtually all plant environmental signals initiate [Ca^2+^]_i_ transients. It is highly likely that there is a complex structural organization on this lipid raft platform that transfers information through the first few steps of transduction and learning. The following are potential constituents of this transduction structure.

A tissue- or cell-specific calcium channel.^163^ Plasma membrane-located calmodulin.^164^ A specific CDPK(s), Asi et al.^165^ Phospholipase C.^166^ A receptor-like protein kinase (RLPK) that acts upstream of MAP kinase cascades^167^ and phosphorylates changes in MAP kinase cascades that lead to ERK 2.^168^ (ERK2 enters the nucleus initiating changes in gene expression, a common response to calcium signals). A plasma membrane-bound Ca^2+^ATPase, activated by calmodulin.^169^ PIP2 is highly negatively charged and located on the inner plasma membrane leaflet. It is electrostatically attracted to membrane regions where Ca^2+^ (positive charge) enters. PIP2 accumulates briefly, attracting proteins that act as initiation sites for actin polymerization (the actin refit) before being hydrolyzed by phospholipase C to IP_3_ that mobilizes Ca^2+^ from the endoplasmic reticulum.

Calcium waves^85-88^ are common results of cellular activation of [Ca^2+^]_I_. They were discussed briefly in “Cell signaling as a cognitive process” and represent the continuation of information transfer leading to tissue learning.

MEMORY. *“Memory can be defined as the recording of experiences that can modify behavior. This general definition encompasses numerous phenomena from the process by which bacteria sense chemical gradients to cognitive learning in humans.”* ^170^ Another definition of memory, equally general, is *the persistence of cellular behaviors after disappearance of their initial cause.* ^171^ Learning and memory are also discussed in^12^ (p. 211–216).

The following indicate five, possibly six different categories of plant memory with illustrated examples only. A complete compilation would necessitate a book. Some, or even all, maybe tissue specific.

(1) **Hebbian-style constructed memory.** Hebb^172^ suggested memories in nervous systems could be formed by increasing the strength of synaptic interactions between neurons that fire together. Such an integration would greatly increase the rate of information flow through this part of the nervous system. Retention of this neural pathway acts as a memory or engram too.

In molecular terms, an equivalent suggestion is to increase, by synthesis, the numbers of rate- constraining proteins and enzymes in a transduction pathway. The consequence would be either a more rapid or a greater transfer of information to the response concerning stressful conditions.^16^ The length of memory in this case would be determined by the stability of the synthesized proteins. The expression of six proteins (CaM, PLC, Ca^2+^-ATPase, CPK, CaN, RLPK) were increased by an estimated order of magnitude from signals such, as touch, osmotic stress, salt stress, low temperature.^17^ More recently, a number of members of the MAPK pathway can be added to the list; receptor protein kinase, MKK1, MEKK1, MKK2, MPK4, MPK6PK19, and two CDPK's and PK19.^173-178^

Whether these proteins are expressed in the same tissues has not been determined, but the data are highly suggestive of a Hebbian-type learning situation. Out of 1000 protein kinases, about 100 are found in the plant MAPK complex. MAPK cascades, crucial to plant memory and resulting in chromatin modification, are reviewed in Sun and Zhang.^179^ A diversity in relaxation time scales of MAPKs can create an emergent transduction memory as indicated above.^180^ Persistent adaptive dynamics in cascades can arise from the diversity of turnover timescales.

(2) **Prions.** Prion proteins provide an unusual method of constructing memory.^181^ A conformational change in the prion mother protein enables it to initiate similar changes in other daughter prion proteins that control processes elsewhere in the cell. The presence of prions can have substantial phenotypic consequences after they are transferred to other cells via mitoses. The changes may be beneficial, benign, or detrimental and could contribute to initiating evolutionary changes. Garai et al.^182^ uncovered a network of prion-like proteins in rice that were both differentially synthesized and regulated in both stress and developmental changes. This mechanism might be responsible for linking stress and memory pathways constructed by genome changes. Interactions with the chaperone Hsp 90 could increase the range of variation.^183^ Prions in animal brains do switch on long term memory by initiating the synthesis of specific proteins (CPEB 3) and this suggests an analogous potential for prion-driven memory in plants.

(3) **Changes in chromatin topology and architecture construct long term stress resistance memories initiated via MAPK and cytosolic Ca^2+^.**

Signal transduction systems in both plant and animal cells share the fundamental mechanisms. In both organism groups, these create networks that are highly cross-linked networks of interacting protein species based around calcium, cyclic nucleotides, MAPK pathways, and other interacting protein kinases. Emergent properties will surely be a consequence and different between animals and plants. Virtually every stress that affects plants generates transients of cellular calcium.^184^ Slight inhibition of the Ca^2+^transient can abolish transcription of mRNAs for proteins specific to the stress.^19^ ^,^ ^20^ Increasing [Ca^2+^]_I_ activates the cross-linked MAPK pathway too (Cargnello and Roux),^185^ possibly via CDPKs causing Raf kinase dimerization.^186^ MAPKK acts as a cytoplasmic anchor of MAPK, but phosphorylation of MAPK by MAPKK activates ERK2 (extracellular signal-regulated kinase) that moves into the nucleus (Lavoie et al).^168^ Chromatin architecture is remodeled from changes in PRC2 (polycomb repressor complex), phosphorylation of RNA polymerase, and modification of histone H3.^187^

Memory of stressful conditions in plants via chromatin architectural change has now been frequently reported.^21^ ^,^ ^188^ ^,^ ^189^ Although originally identified as priming, the mechanism used is similar to those in animal cells, including nerve cells (e.g.^190^ ^,^ ^191^). Touch and wind are mechanical signals which elevate cytosolic and nuclear calcium in plant cells.^20^ Recent data show that the consequent mechanical deformation of the nucleus modifies both nuclear and cytoplasmic behavior.^148^

(4) **Potential electrical memories**

There are a number of interesting examples that suggest electrical memory may be the basis of the observations. Viner et al.^192^ investigated leaf unrolling in dark-grown barley leaves. Initiation of leaf unrolling is normally instigated by the generation of 5 minutes of Pfr. EGTA inhibits unrolling, but addition of Ca^2+^ can initiate unrolling only if given within one hour of the generation of Pfr. Another red-light signal, together with added Ca, given four hours after the initial red light, immediately initiates unrolling. Pfr normally institutes changes in membrane potential and ion channel activity.

For tendril coiling in *Pisum*, two signals are required: touch and blue light. If the tendril is touched in darkness, the blue light irradiation can be delayed for up to two hours.^193^ Touch normally induces changes in membrane potential and entry of Ca^2+^ with potential secondary influences. Which blue light receptors are needed is not known, but much early work identified changes in membrane potential and redox activity implying electron flow.

A hypo-osmotic shock to cultured cells induces a calcium transient. If extracellular calcium is absent, the transient is not generated but can appear if extracellular calcium is added within 20 minutes of the initial shock.^194^ All these events need further research to better understand them and see if they are electrically based.

Volkovet al.^195^ indicate electrical memory in the Venus flytrap, a trap which is sprung by electrical and [Ca^2=^]_i_ changes and has an accumulated memory requiring five touches of the trap hair to initiate digestion and only two to close the trap.^196^

(5) **Transduction system memory**

In the introduction to this article, it was indicated that Bhalla and Iyengar^22^ reported that memory was one emergent property of signal transduction. Another article has identified an emergent memory in cell signaling but more clearly specified Mitra et al.^180^ These authors concentrated on the MAP kinase pathway and reported an abundant repertoire of transient adaptive responses with long-lived modulations in frequency. On withdrawing the stimulus to the pathway, the pathway decays only over long-time scales. The MAP kinase cascade retains a memory as well as identifying its central role in information processing by animal and plant cells.

Very brief exposure of dark-grown coleoptiles to a directional blue light stimulus can be overridden by a blue light stimulus from the opposite side, provided it is given in less than an hour after the first. A memory is instituted by the first signal in the form of a labile precursor. A memory is instituted by the first signal in the form of a labile precursor. This memory is truncated by the formation of microtubules oriented in a longitudinal direction after 60 minutes in cells of the outer epidermis.^197^ ^,^ ^198^ But if the second irradiation is delayed until 90 minutes or later, the initial irradiation is no longer overridden. Microtubule formation is likely initiated and controlled by spatially-localized generation of cyclic nucleotides (along with an equally located phosphodiesterase and gamma tubulin acting as a microtubule nucleation site Kiwiatowski et al.^199^).

### The uninvestigated: biophotons and electromagnetic fields

One polarizing signal impacting on the *Fucus* zygote are the near presence of other seaweeds. Jaffe^200^ reported that the seaweed signal is not blocked by an intervening glass slide. He concluded that luminescent light, biophotons, was the polarizing signal. Albrecht-Buehler^201^ reported that a culture of cells whose axes were oriented by infrared light when placed behind a glass screen, oriented others whose initial axes were random. Metallizing the glass screen prevented communication. He described it as primitive vision from infrared radiation emitted by the axes-oriented cells.

Biophotons or ultra-weak photon emission (UWPE) are usually characterized as photon fluxes below 10^4^ photons cm^−2^ .s^−1^.^202^ All living organisms have been found to emit them, they may, in part, result from cell oxidative reactions and increase in amount under stressful conditions. The introduction of photomultipliers has demonstrated the universal nature of UWPE.^203^ The critical result of Jaffe's^200^ paper is not just the conclusion of biophotons synthesized by other seaweeds but that they initiated a biological response in *Fucus* development. Does this perception require a specific receptor?

UWPE has a broad spectrum of visible light, but simple calculation indicates its intensity is well below the fluence response of the most highly sensitive blue light-initiated phototropism in bean.^204^ But it is approximately within the range of the very low fluence response of phytochrome in lettuce seed.^205^ But red light does not polarize the *Fucus* zygote, Jaffe.^206^ Jaffe^200^ considered the seaweed biophotons to be concentrated in the red part of the spectrum. A common theme in the literature on biophotons is an association of UWPE with microtubule activities and function, which is a later part of axis formation.^200^ ^,^ ^203^ Jaffe exposed his *Fucus* zygotes continuously to UWPE and given the very low density of UWPE, this suggests the need for a cumulative memory storing information within the developmental window that finally initiates the program leading to the first asymmetric division.

Light is of course electromagnetic, but visible light covers only one narrow range of electromagnetic radiation. In the past, many different electrical wavelengths have been shown to be both generated and sensed by cells and organisms.^203^ The Book “Fields of the Cells” edited by Fels, Cifra, and Scholkmann,^207^ is free to download on the web and contains 16 chapters that cover many aspects of this large research area.

Investigations of electromagnetic signal synthesis, transduction, memory, and response of radiation beyond the visible are simply absent in plants. But two papers and a book by^208^ ^,^ ^209^ indicate the potential in this area. It is commonly supposed that morphogenesis of new developing tissues requires the translocation of specifically synthesized information to the correct place and at the correct time and within a developmental window. As Pietack points out, a dynamic system in a self-developing organ can self-trap electromagnetic energy that acts as a symmetry-breaking mechanism informing on cell position, polarization, orientation, genetic expression, and differentiation state. Pietack ^208^ ^,^ ^209^ demonstrates this beautifully for the construction of an electromagnetic field that mimics the eventual distribution of cell types and internal structure in the formation of the cucurbit ovary and for vascular tissue distribution in differently-shaped leaves. The 2009 paper is expanded in Chapter 5 of her book Pietack.^210^ These patterns develop amongst a mass of undifferentiated dividing cells, but the field pattern is determined by the developing shape of the organ.

### Summary of learning and memory

I have indicated five examples of memory. Memory can only be acquired if it is learnt in some way. The use of these plant memories by plants needs investigation to understand how widespread their memory basis is. Memory three, chromatin changes, has received some characterization, but the detailed mechanisms that lead to modifications of chromatin structure need proper characterization in plant systems. Plant memories are currently an open field for investigation. They need better characterization, not only for plants in many environmental circumstances but for crop development too. The full panoply of memory and learning should surely be used by plants in wilderness conditions. Investigations of that potential would require taking molecular investigations into wilderness. Adaptive responses acting as intelligence behavior would then receive much greater understanding. While recognizing the difficulties of so doing, the rewards in real understanding would surely be legion. The laboratory might be convenient at present, but concentration on taking molecular studies into natural circumstances is the logical way forward. There will be abundant new and exciting times to come if this particular road is followed.

No grant supported this article. I am grateful to the suggestions made by five referees. They have greatly improved the quality of the manuscript.
